# Inflammatory cytokine and chemokine profiles are associated with patient outcome and the hyperadrenergic state following acute brain injury

**DOI:** 10.1186/s12974-016-0500-3

**Published:** 2016-02-16

**Authors:** Alex P. Di Battista, Shawn G. Rhind, Michael G. Hutchison, Syed Hassan, Maria Y. Shiu, Kenji Inaba, Jane Topolovec-Vranic, Antonio Capone Neto, Sandro B. Rizoli, Andrew J. Baker

**Affiliations:** Defence Research & Development Canada, Toronto Research Centre, Toronto, ON Canada; Institute of Medical Science, University of Toronto, Toronto, ON Canada; Faculty of Kinesiology & Physical Education, University of Toronto, Toronto, ON Canada; Keenan Research Centre, Li Ka Shing Knowledge Institute, St. Michael’s Hopsital, Toronto, ON Canada; Division of Trauma & Critical Care, University of Southern California, Los Angeles, CA USA; LA County+ USC Medical Center, Los Angeles, CA USA; Department of Critical Care, St. Michael’s Hospital, University of Toronto, Toronto, ON Canada; Department of Anesthesia, St. Michael’s Hospital, University of Toronto, Toronto, ON Canada; Department of Surgery, St. Michael’s Hospital, University of Toronto, Toronto, ON Canada; Institute for Healthcare Improvement (IHI) Fellow, Cambridge, MA USA

**Keywords:** Catecholamines, Traumatic brain injury, Systemic inflammation, Sympathetic nervous system activation, Epinephrine, Norepinephrine, IL-10, IL-1β

## Abstract

**Background:**

Traumatic brain injury (TBI) elicits intense sympathetic nervous system (SNS) activation with profuse catecholamine secretion. The resultant hyperadrenergic state is linked to immunomodulation both within the brain and systemically. Dysregulated inflammation post-TBI exacerbates secondary brain injury and contributes to unfavorable patient outcomes including death. The aim of this study was to characterize the early dynamic profile of circulating inflammatory cytokines/chemokines in patients admitted for moderate-to-severe TBI, to examine interrelationships between these mediators and catecholamines, as well as clinical indices of injury severity and neurological outcome.

**Methods:**

Blood was sampled from 166 isolated TBI patients (aged 45 ± 20.3 years; 74.7 % male) on admission, 6-, 12-, and 24-h post-injury and from healthy controls (*N* = 21). Plasma cytokine [interleukin (IL)-1β, -2, -4, -5, -10, -12p70, -13, tumor necrosis factor (TNF)-α, interferon (IFN)-γ] and chemokine [IL-8, eotaxin, eotaxin-3, IFN-γ-induced protein (IP)-10, monocyte chemoattractant protein (MCP)-1, -4, macrophage-derived chemokine (MDC), macrophage inflammatory protein (MIP)-1β, thymus activation regulated chemokine (TARC)] concentrations were analyzed using high-sensitivity electrochemiluminescence multiplex immunoassays. Plasma catecholamines [epinephrine (Epi), norepinephrine (NE)] were measured by immunoassay. Neurological outcome at 6 months was assessed using the extended Glasgow outcome scale (GOSE) dichotomized as good (>4) or poor (≤4) outcomes.

**Results:**

Patients showed altered levels of IL-10 and all chemokines assayed relative to controls. Significant differences in a number of markers were evident between moderate and severe TBI cohorts. Elevated IL-8, IL-10, and TNF-α, as well as alterations in 8 of 9 chemokines, were associated with poor outcome at 6 months. Notably, a positive association was found between Epi and IL-1β, IL-10, Eotaxin, IL-8, and MCP-1. NE was positively associated with IL-1β, IL-10, TNF-α, eotaxin, IL-8, IP-10, and MCP-1.

**Conclusions:**

Our results provide further evidence that exaggerated SNS activation acutely after isolated TBI in humans may contribute to harmful peripheral inflammatory cytokine/chemokine dysregulation. These findings are consistent with a potentially beneficial role for therapies aimed at modulating the inflammatory response and hyperadrenergic state acutely post-injury.

**Electronic supplementary material:**

The online version of this article (doi:10.1186/s12974-016-0500-3) contains supplementary material, which is available to authorized users.

## Background

Growing experimental and clinical evidence indicates that inflammation is an integral component to the pathogenesis of secondary injury after traumatic brain injury (TBI) [1, 2]. Its effects are not limited to the brain parenchyma, as systemic inflammation is a noted consequence of TBI, and can impact patient outcome by exacerbating cerebral tissue injury and contributing to systemic complications such as nosocomial infection and multiple organ failure [2–4]. While potentially harmful, bi-directional neuroimmune communication between the central nervous system (CNS) and the periphery is essential for neuronal repair and regeneration [5]. The duality of this process is highlighted by a number of failed clinical trials aimed broadly at reducing or inhibiting inflammation [6]. Hence, a better understanding of the underlying mechanisms governing the inflammatory response to isolated TBI in humans can help guide future therapeutic strategies and improve patient outcome.

In an effort to restore vital homeostasis in the face of TBI, activation of the sympathetic nervous system (SNS) results in a massive secretion of catecholamines [epinephrine (Epi), norepinephrine (NE)] into the periphery as part of the generalized host stress response to trauma [7–13]. Moreover, we have recently demonstrated in a large group of moderate-to-severe TBI patients, that both NE and Epi are elevated in a dose-dependent fashion according to injury severity, and that prolonged elevation of NE and Epi throughout the first 24 h after hospital admission is highly correlated with adverse patient outcomes [14].

It has been hypothesized that early SNS activation after TBI may influence the inflammatory response both locally and systemically. This occurs prototypically as a response to elevations in cerebral IL-1β concentrations and subsequent initiation of both the local and systemic acute phase response [4, 15–18]. In addition, trauma-induced activation of NE terminals in peripheral organs such as the liver and spleen may lead to the systemic release of inflammatory mediators into the circulation [4, 19, 20]. It is also possible that elevated concentrations of peripheral catecholamines can differentially alter cytokine/chemokine production in circulating lymphocytes [21]. NE and Epi interact with α- and β-adrenergic receptors expressed on leukocytes and other tissues, influencing the production of inflammatory mediators from these cells [22–25]. Furthermore, β-blocker therapy in human TBI has been associated with improved outcome [26, 27], and a number of animal studies using pharmacological blockade of β-adrenergic receptors have shown concomitant attenuation of the inflammatory response and improved outcome after treatment [28–30]. However, no previous clinical studies have evaluated the relationship between the SNS and systemic inflammation in isolated TBI patients.

Elevations in a number of cytokines, including interleukin (IL)-1β, -6, -10, and TNF-α, have been identified in the circulation of TBI patients within hours of injury [31–36]. However, their relationship to patient survival and outcome is less clear, likely owing to the heterogeneous nature of both primary brain injury (i.e., focal vs. blunt trauma and extracranial complications) and the complexity of secondary injury processes. Additionally, correlations have been found between early elevations in peripheral cytokines and poor patient outcome [37–40], although others have found no association [34, 41] or have even identified inverse relationships between cytokines and risk of infection after injury [42]. Moreover, chemokines appear to play an important role in TBI pathophysiology [43], though human studies to date have focused predominantly on IL-8 and MCP-1 [39, 44, 45] and require further characterization.

Therefore, the purpose of this study was to (1) identify the temporal profile of a panel of circulating cytokines and chemokines acutely after injury in both moderate and severe isolated blunt TBI patients; (2) identify possible interrelationships between circulating catecholamines and cytokines/chemokines post-injury; (3) to evaluate these markers in patients stratified according to 6-month neurological outcome and mortality.

## Methods

### Ethics statement

The study protocol complied with the ethical guidelines of the Declaration of Helsinki of 1975 and was approved by the Research Ethics Boards and Institutional Review Boards of the participating hospitals. All patients’ families received a comprehensive description of the study and gave written informed consent for their relatives’ participation. In the absence of a substitute decision-maker, consent was delayed in accordance with the Tri-Council Policy Agreement for Research in Emergency Health Situations (Article 2.8); delayed written consent for participation in the study was subsequently obtained from next-of-kin or, where possible, directly from the patient once they had recovered sufficiently. Informed consent for a single blood sample was also obtained from healthy (control) volunteers.

### Patients and controls

Potential study participants were admitted to Sunnybrook Health Sciences Centre (Toronto, ON, Canada), St. Michael’s Hospital (Toronto, ON, Canada), and Los Angeles County + University of Southern California (USC) Medical Center (Los Angeles, CA). Upon admission, patients with an isolated TBI, defined by a Glasgow coma scale (GCS) score of <13 and a non-head abbreviated injury score (AIS) of ≤2, were considered for inclusion. Patients with an elapsed time between trauma and hospital admission of >3 h, with a penetrating brain injury, <16 years of age, pregnant, taking β-blockers, lacking vital signs prior to admission, or clinically brain dead on admission were excluded. A healthy control group with no history of brain injury was included for a single blood donation for analysis of the selected panel of soluble inflammatory markers.

### Study design and procedures

Upon hospital admission, clinical and demographic data were obtained from eligible patients: *demographics—*age and sex ; *clinical information—*mechanism of injury, elapsed time from the trauma to the emergency room, injury severity score (ISS), and AIS head; *neurological status—*level of consciousness categorized by the GCS, pupil size and reactivity, seizures, and alcohol level; *clinical status—*blood pressure, tracheal intubation, spontaneous vs. mechanical ventilation, oxygen saturation, and temperature; and *medical history*—past medical history, present medications including β-blockers and anticoagulants. Routine laboratory exams and imaging were also completed upon admission, including chest radiography and computerized tomography (CT) scans. All significant clinical events during the first 24 h were recorded, including, but not limited to, sepsis/infection, organ failure, any medical treatments administered, surgical procedures, and any other significant changes in clinical parameters. Organ failure was defined by the following criteria: (1) arterial hypoxemia—PaO_2_/FiO_2_ < 300; (2) acute oliguria—urine output <0.5 mL/kg/h for at least 2 h despite adequate fluid resuscitation; (3) creatine increase—>0.5 mg/dL or 44.2 μmol/L; (4) coagulation abnormalities—INR > 1.5 or aPTT > 60 s; (5) ileus—absent bowel sounds; (6) thrombocytopenia—platelet count <100,000 μ/L; and (7) hyperbilirubinemia—plasma total bilirubin >4 mg/dL or 70 μmol/L.

For patients who died, the cause of death was recorded and classified as TBI-related or non-TBI related. Upon hospital discharge, at 28 days and at 6-months, patient outcome was assessed using the extended Glasgow outcome scale (GOSE)  .

### Blood sample collection

Venous blood samples were drawn as soon as possible after admission to the trauma room or emergency department, and again at 6-, 12-, and 24-h post-injury. Samples were drawn into either 10 mL K_2_EDTA [with 4 mM sodium metabisulfite (Na_2_S_2_O_5_)] or 10-mL sodium heparin vacutainers (Vacutainer, Becton Dickinson, Rutherford, NJ). Specimens were immediately centrifuged at 1600×*g* for 15 min at 4 °C; the plasma supernatant was then separated into six (1–2 mL) aliquots and frozen at −70 °C until subsequent analysis.

### Multiplex cytokine and chemokine measurements

Immunoreactive plasma levels of selected cytokines and chemokines were analyzed with Meso Scale Discovery (MSD) 96-Well MULTI-SPOT^®^ Ultra-Sensitive Human Immunoassay Kits, using electrochemiluminescence detection on an MSD Sector Imager^™^ 6000 with Discovery Workbench software (version 3.0.18) (MSD^®^, Gaitherburg, MD, USA). Cytokines were measured using the TH1/TH2 10-plex kit, which included nine markers (excluding IL-8): IFN-γ, IL-1 β, -2, -4, -5, -10, -12p70, -13, and TNF-α. Chemokines were measured using the Chemokine 9-plex, which also included nine markers: eotaxin, eotaxin-3, MIP-1β, thymus activation regulated chemokine (TARC), IP-10, IL-8, MCP-1, MDC, and MCP-4 (Additional file 1: Table S1). All assays were performed according to manufacturer’s instructions, in duplicates, and without alterations to the recommended standard curve dilutions. Briefly, samples were thawed on ice and added to a 96-well MULTI-SPOT^®^ plate coated with capture antibodies in a spatially distinct fashion. SULFO-TAG^®^ labeled detection antibodies were then added to each of the wells to complete the sandwich format, and a read buffer was added to alter the chemical environment for electrochemiluminescence. The subsequent reaction resulted in the emission of light from the labeled analytes, which was then quantified to approximate the concentration (pg/mL) of each protein present in the sample.

### Catecholamine measurements

Plasma Epi and NE levels were determined from duplicate samples using a competitive enzyme immunoassay method according to the manufacturer’s instructions (Bi-CAT EIA, ALPCO Diagnostics, Salem, NH). Briefly, plasma Epi and NE were extracted using a cis-diol-specific affinity gel, acylated and then derivatized enzymatically into *n*-acylmetanephrine and *n*-acylnormetanephrine, respectively. Antibody bound to the solid-phase catecholamines were detected by an anti-rabbit IgG-peroxidase conjugate using tetramethylbenzidine as a substrate. Quantification of unknown samples was achieved by comparing their absorbance with a reference curve prepared with known standard concentrations included in the kit.

### Statistical analysis

Demographic and clinical parameters are expressed as the mean ± standard deviation (SD) unless otherwise stated, while blood marker concentrations are graphically displayed as the median and interquartile range. Comparison of inflammatory marker levels between TBI patients at each sampled time point and healthy control subjects was performed using a Kruskal-Wallis analysis of variance, with Dunn’s multiple comparisons post hoc test. To assess 6-month neurological outcome, patients were dichotomized into favorable (GOSE 5–8) and unfavorable (GOSE 1–4) outcomes. Mortality was assessed by stratifying patients into two groups, “survivors” and “non-survivors.” Death was further stratified as either “neurologic” or “non-neurologic.” Differences in cytokine and chemokine concentrations between moderate and severe TBI patients, unfavorable and favorable outcome, survivors and non-survivors, and neurologic and non-neurologic death were assessed by Mann-Whitney *U*. To identify possible correlations between catecholamines and inflammatory marker concentrations, pooled data over all time points was evaluated by Spearman’s *ρ*. To generate an aggregate inflammation score (IS), peak quartile rank scores from each marker associated with both unfavorable outcome and survival were summed [36, 46]. For each marker, values >75th percentile were given a score of 4, values between the 50th and 75th percentile were given a score of 3, values between the 25th and 50th percentile were given a score of 2, and values <25th percentile were given a score of 1. When lower concentrations of a marker were associated with unfavorable patient outcome, the scoring system was reversed, hence values <25th percentile were given a score of 4, while values >75th percentile were given a score of 1. Quartile scores of each marker were added, resulting in an aggregate score; six markers were included, allowing for a score ranging from 6 to 24. Patients were then dichotomized into high- vs. low-inflammation categories based on the median aggregate IS [47]. In addition, to assess the ability of cytokines and chemokines to predict poor patient outcome and death while controlling for injury severity, a multivariate binomial logistic regression analysis was employed. All markers were quartiled in order to standardize unit increases for statistical comparison. Each individual marker was added independently to a model containing GCS and AIS head scores. The binary dependent outcome variables were 6-month GOSE or survival. Biomarker data was not statistically analyzed or graphically displayed unless at least 50 % of the samples analyzed were within the detection range of the assay, or contained replicate values with a coefficient of variation (CV) <25 %. Statistical significance in all analyses was indicated by a *p* value of ≤0.05. All data were analyzed using GraphPad Prism Version 6.0d (GraphPad Inc, CA, USA) and Stata Version 13.1 (StataCorp, TX, USA).

## Results

### Demographics and clinical characteristics

Table 1 summarizes the demographic, clinical, and outcome data for the 166 (33 moderate, 133 severe) patients analyzed in the study. Subjects were predominantly male (*n* = 124, 74.7 %), with an average age of 45.8 ± 20.3 years . The majority of patients had an unfavorable outcome at 6 months, classified as a GOSE score of 1–4 (*n* = 102, 61.4 %). Eighteen patients (10.8 %) developed organ failure, and there were 45 (27.1 %) deaths. Among the 21 healthy control subjects, 71.4 % were males (*n* = 15), and the mean age was 32.7 ± 7.8 years (data not shown).

**Table 1:** Demographic and clinical characteristics for TBI patients

Characteristics	All TBI patients (*n* = 166)	Moderate TBI (*n* = 33)	Severe TBI (*n* = 133)
Demographics			
Age (years)	45.8 ± 20.3	49.5 ± 19.7	44.9 ± 20.4
Male —*n* (%)	124 (74.7)	23 (69.7)	101 (75.9)
Clinical characteristics			
Time to ED (min)	77.6 ± 63.5	74.7 ± 64.8	78.3 ± 63.4
ISS score	24.4 ± 11.8	18.8 ± 11.5	25.9 ± 11.5
AIS head	4.1 ± 1.1	3.5 ± 1.2	4.3 ± 1.0
GCS	5.9 ± 3.0	10.5 ± 1.3	4.7 ± 1.9
Marshall score—*n* (%)			
I	28 (16.9)	11 (33.3)	17 (12.8)
II	76 (45.8)	16 (48.5)	60 (45.1)
III	13 (7.8)	1 (3.0)	12 (9.0)
IV	27 (16.3)	3 (9.1)	24 (18.0)
Evacuated mass lesion	21 (12.6)	2 (6.1)	19 (14.3)
Non-evacuated mass lesion	1 (0.6)	0 (0.0)	1 (0.7)
Pre-injury comorbidities—*n* (%)	45 (27.1)	14 (42.4)	31 (23.3)
Outcomes			
Mortality*—n* (%)	45 (27.1)	1 (3.0)	44 (33.0)
Neurosurgery performed—*n* (%)	47 (28.3)	5 (15.1)	42 (31.6)
Sepsis/infection—*n* (%)	42 (25.3)	7 (21.2)	35 (26.3)
Organ failure—*n* (%)	18 (10.8)	0 (0.0)	18 (13.5)
6-month GOSE	4.0 ± 2.5	5.7 ± 2.1	3.6 ± 2.5

Unless otherwise stated, results are expressed as mean ± standard deviation (SD)

*Abbreviations*: *TBI* traumatic brain injury, *ED* emergency department, *ISS* injury severity score, *AIS* abbreviated injury scale, *GCS* Glasgow coma scale, *GOSE* extended Glasgow outcome scale

### Plasma concentrations of inflammatory markers in moderate and severe TBI patients

**Fig. 1:**
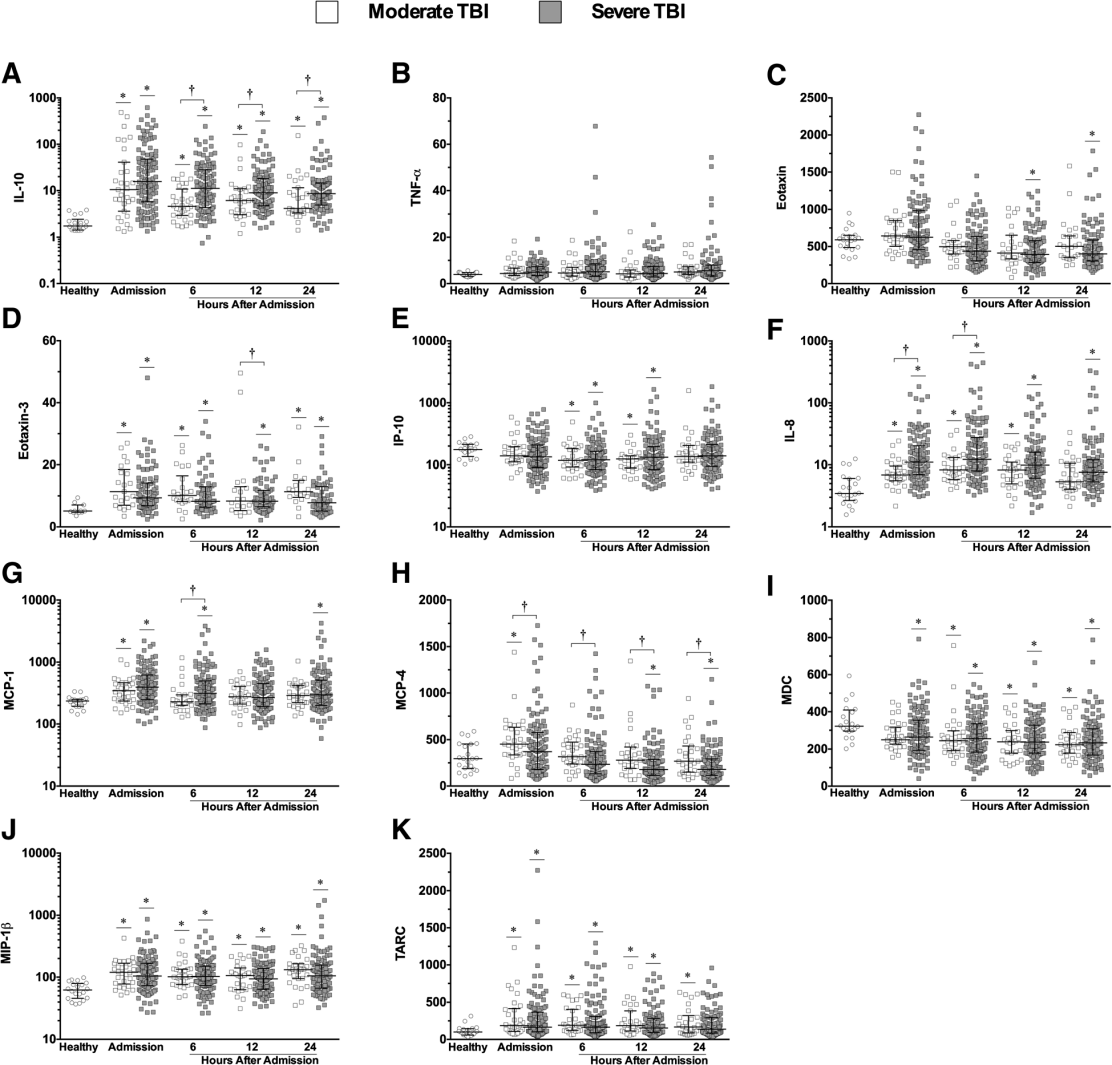
Plasma cytokine and chemokine concentrations in moderate and severe TBI patients sampled over 24 h. Cytokines interleukin (*IL*)-10 (**a**), tumor necrosis factor (*TNF*)-α (**b**). Chemokines eotaxin, eotaxin-3, interferon-gamma induced protein (*IP*)-10, IL-8, monocyte chemoattractant protein (*MCP*)-1, -4, macrophage-derived chemokine (*MDC*), macrophage inflammatory protein (*MIP*)-1β, and thymus and activation regulated chemokine (*TARC*) (**c**–**k**) in moderate (GCS 9–12, *n* = 33, *open squares*) and severe (GCS 3–8, *n* = 133, *closed squares*) TBI patients within the first 24 h of hospital admission vs. healthy control subjects (no TBI, *n* = 21, *open circles*). *Lines* represent the median and interquartile range. *p < 0.05 vs. healthy controls by Kruskal-Wallis. ^†^ *p* < 0.05 vs. moderate TBI by Mann-Whitney *U* test

Plasma concentrations of cytokines and chemokines stratified according to moderate (GCS 9–12) and severe (GCS 3–8) TBI are shown in Fig. 1. IL-10 was the only cytokine altered in patients compared with healthy controls—median admission IL-10 levels were 5 - and 9 - fold higher, respectively, in moderate and severe TBI patients, and were significantly elevated at all time points (Fig. 1a). Furthermore, at 6, 12, and 24 h after hospital admission, mean IL-10 levels in severe TBI patients were significantly elevated (~2-fold at admission, 6, and 24 h) compared to moderate TBI patients (Fig. 1a).

Significant alterations in all nine chemokines were detected in TBI patients dichotomized by injury severity vs. healthy controls (Fig. 1c–k). The most dramatic increase was seen in IL-8: patient concentrations peaked at 6 h after hospital admission and were nearly 3.5-fold higher in those with severe TBI vs. healthy control subjects (median concentrations, 12.2 vs. 3.5 pg/mL, respectively) (Fig. 1f). In addition, at admission and 6 h, mean IL-8 levels were higher in patients with severe TBI compared to those with moderate TBI (Fig. 1f). Significant increases in MCP-1 concentrations in severe vs. moderate TBI patients were also identified (6 h) (Fig. 1g). Conversely, IP-10 and MDC concentrations were decreased in both moderate and severe TBI patients compared with controls (Fig. 1e, i, respectively), while eotaxin was decreased in severe TBI patients only (Fig. 1c). MCP-4 levels were elevated on admission in moderate TBI patients compared with healthy subjects, but were lower in severe TBI patients compared with healthy subjects at 12 and 24 h (Fig. 1h). Furthermore, MCP-4 levels were lower in severe TBI patients at all sampled time points compared to moderate TBI patients (Fig. 1h). Eotaxin levels were elevated in severe TBI patients at admission, but similar to MCP-4, were decreased at 12 and 24 h (Fig. 1c).

### Correlation between SNS and inflammatory markers

Pooled concentrations of NE and Epi were associated with a number of inflammatory markers acutely after TBI (Table 2). IL-1β and IL-10 were positively correlated to Epi, with IL-10 displaying the strongest correlation (*r* = 0.44, *P <* 0.01) (Table 3). NE concentrations were positively related to IL-1β, IL-10, and TNF-α (Table 2). Similar to Epi, NE was most strongly correlated to IL-10 (*r* = 0.45, *P <* 0.01). In addition, IL-1β displayed a stronger positive association with NE (*r* = 0.28, *P <* 0.01) than with Epi (*r* = 0.11, *P <* 0.03) (Table 2).

**Table 2:** Catecholamine and inflammatory marker correlations

Marker	Catecholamines			
	Epinephrine		Norepinephrine	
	*r*	*P* value	*r*	*P* value
Cytokines (pg/mL)				
IL-1β	0.11	0.04^*^	0.28	<0.01^*^
IL-5	−0.07	0.19	0.01	0.90
IL-10	0.44	<0.01^*^	0.45	<0.01^*^
TNF-α	0.00	0.93	0.14	<0.01^*^
Chemokines (pg/mL)				
Eotaxin	0.16	<0.01^*^	0.16	0.01^*^
Eotaxin-3	0.08	0.15	0.05	0.31
IL-8	0.35	<0.01^*^	0.39	<0.01^*^
IP-10	−0.00	0.88	0.10	0.02^*^
MCP-1	0.23	<0.01^*^	0.33	<0.01^*^
MCP-4	0.06	0.17	0.05	0.24
MDC	−0.00	0.98	−0.09	0.04^*^
MIP-1β	−0.00	0.97	0.16	<0.01
TARC	−0.05	0.24	0.03	0.43

*Abbreviations*: *IL* interleukin, *TNF-α* tumor necrosis factor-alpha, *IP-10* interferon-gamma induced protein-10, *MCP* monocyte chemoattractant protein, *MDC* macrophage-derived chemokine, *MIP-1β* macrophage inflammatory protein–1β, *TARC* thymus and activation regulated chemokine

*p < 0.05 via Spearman’s *ρ*

**Table 3:** Binomial logistic regression models assessing the ability of inflammatory markers to predict poor patient outcome, controlling for injury severity

Marker	Admission		Hours after admission					
			6		12		24	
	OR	*P* value	OR	*P* value	OR	*P* value	OR	*P* value
Unfavorable 6-month GOSE								
IL-1β	0.84	0.489	1.84^*^	0.002	1.65^*^	0.009	0.91	0.673
IL-10	1.18	0.376	1.76^*^	0.005	1.57^*^	0.019	1.48^*^	0.034
TNF-α	1.49^*^	0.024	1.61^*^	0.008	1.50^*^	0.021	1.30	0.138
IL-8	1.66^*^	0.007	1.48^*^	0.030	1.44^*^	0.036	1.33	0.104
MCP-1	1.42	0.052	1.09	0.603	1.24	0.213	1.39	0.067
MDC	1.10	0.586	0.90	0.515	1.45	0.037^*^	0.85	0.363
Mortality								
IL-1β	1.02	0.935	1.06	0.825	1.85^*^	0.038	1.15	0.598
IL-10	1.19	0.417	2.82^*^	<0.001	2.09^*^	0.003	2.20^*^	0.003^*^
TNF-α	1.58^*^	0.027	1.77^*^	0.007	1.58^*^	0.033	1.19	0.395
IL-8	1.31	0.168	1.89^*^	0.005	1.84	0.010^*^	1.37	0.176
IP-10	3.11^*^	<0.001	2.06^*^	0.002	1.59^*^	0.030	1.19	0.400
MCP-1	1.26	0.230	1.46	0.069	1.57^*^	0.035	0.91	0.674
MCP-4	1.43	0.090	1.49	0.065	0.99	0.964	0.57^*^	0.023
MDC	1.03	0.864	0.91	0.620	1.14	0.529	0.71	0.114
MIP-1β	1.13	0.544	1.27	0.243	1.29	0.221	0.89	0.592
TARC	0.95	0.801	1.00	0.991	0.77	0.231	0.56^*^	0.015
Eotaxin	1.90^*^	0.005	1.90^*^	0.007	1.33	0.208	0.79	0.304

Models were controlled for admission GCS and AIS head scores

All blood biomarker concentrations were standardized by quartiles. A one-unit increase is equivalent to a 25 % increase in biomarker concentration

*Abbreviations*: *OR* odds ratio, *GOSE* extended Glasgow outcome scale, *IL* interleukin, *TNF-α* tumor necrosis factor-alpha, *MCP* monocyte chemoattractant protein, *MDC* macrophage-derived chemokine, *IP-10* interferon-gamma induced protein-10, *MIP-1β* macrophage inflammatory protein–1β, *TARC* thymus and activation regulated chemokine

*p < 0.05

 E otaxin, IL-8, and MCP-1 were positively related to both NE and Epi, while IP-10 was associated with NE only (Table 2). The strongest relationship between catecholamines and chemokines was found between IL-8 and NE (*r* = 0.39, *P <* 0.01); IL-8 also displayed the highest correlation of any chemokine with Epi (*r* = 0.35, *P <* 0.01) (Table 2).

### Six-month neurological outcome

**Fig. 2:**
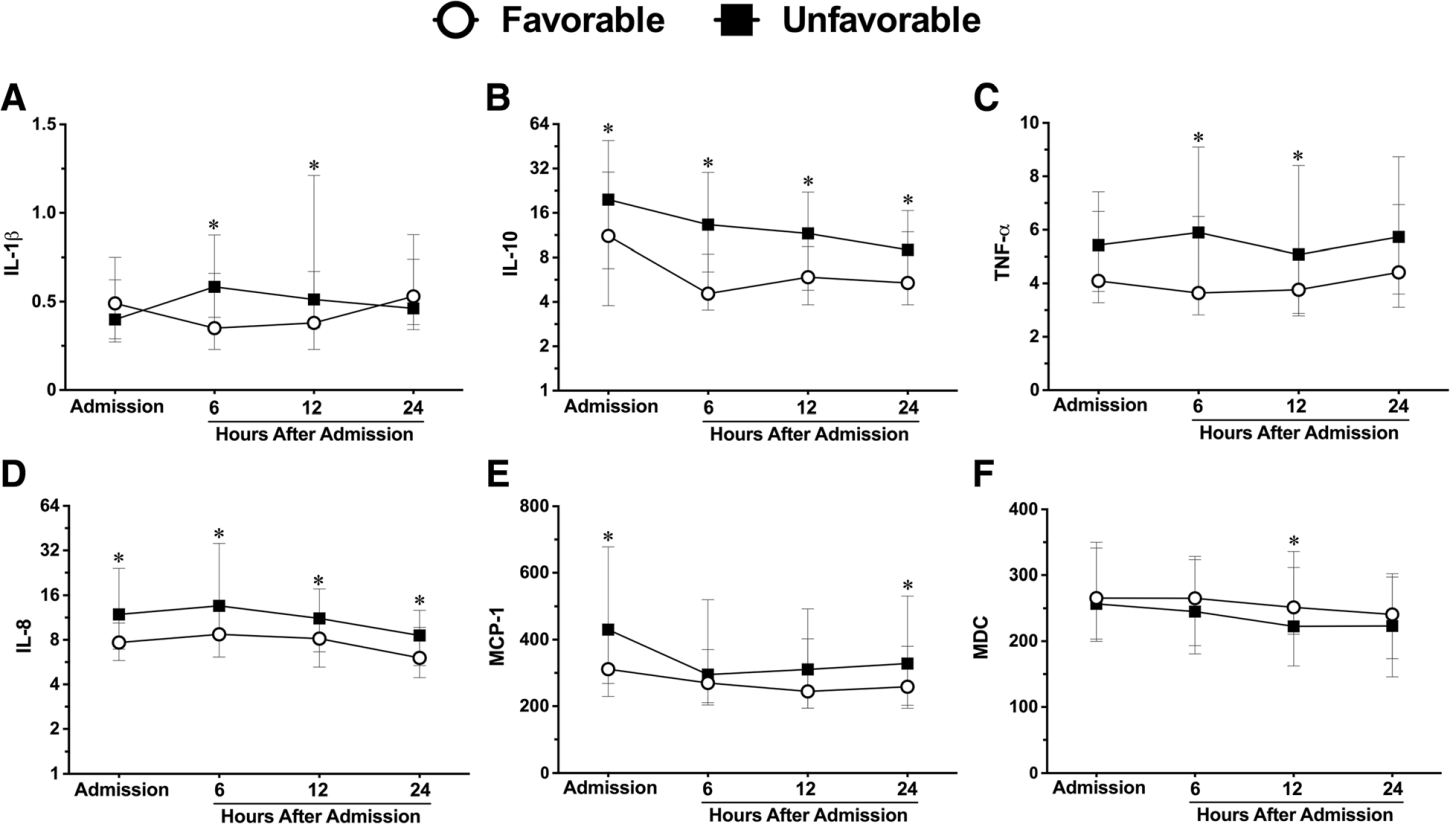
Plasma cytokine and chemokine concentrations in TBI patients stratified according to the 6-month GOSE. I nterleukin (*IL*)-1β, -10, tumor necrosis factor (*TNF*)-α (**a**–**c**). IL-8, monocyte chemoattractant protein (*MCP*)-1, macrophage-derived chemokine (*MDC*) (**d**–**f**) in TBI patients with unfavorable (GOSE 1–4, *n* = 102) vs. favorable (GOSE 5–8, *n* = 61) 6-month neurological outcome. *Lines* represent the median and interquartile range. ^*^ *p* < 0.05 *vs*. favorable outcome by Mann-Whitney *U* test

Within 24 h of hospital admission, significant differences in plasma levels of three cytokines and three chemokines were observed between patients with favorable and unfavorable 6-month neurological outcome (Fig. 2a–f respectively). IL-10 displayed the most profound difference of all cytokines assessed: concentrations at 6 h were near 3-fold higher in patients with unfavorable vs. favorable 6-month outcome (median concentrations, 13.3 vs. 4.5 pg/mL, respectively) and were significantly elevated at all sampled time points (Fig. 2b). In addition, both IL-1β and TNF-α concentrations were elevated at 6 and 12 h in patients with unfavorable vs. favorable outcome (Fig. 2a, c, respectively). IL-8 levels were significantly elevated at all sampled time points, with a peak difference at 6 h (13.6 vs. 8.7 pg/mL in unfavorable vs. favorable outcome, respectively), while MCP-1 concentrations were significantly elevated at admission and 24 h in patients with unfavorable vs. favorable outcome (Fig. 2d, e, respectively).

### Mortality

**Fig. 3:**
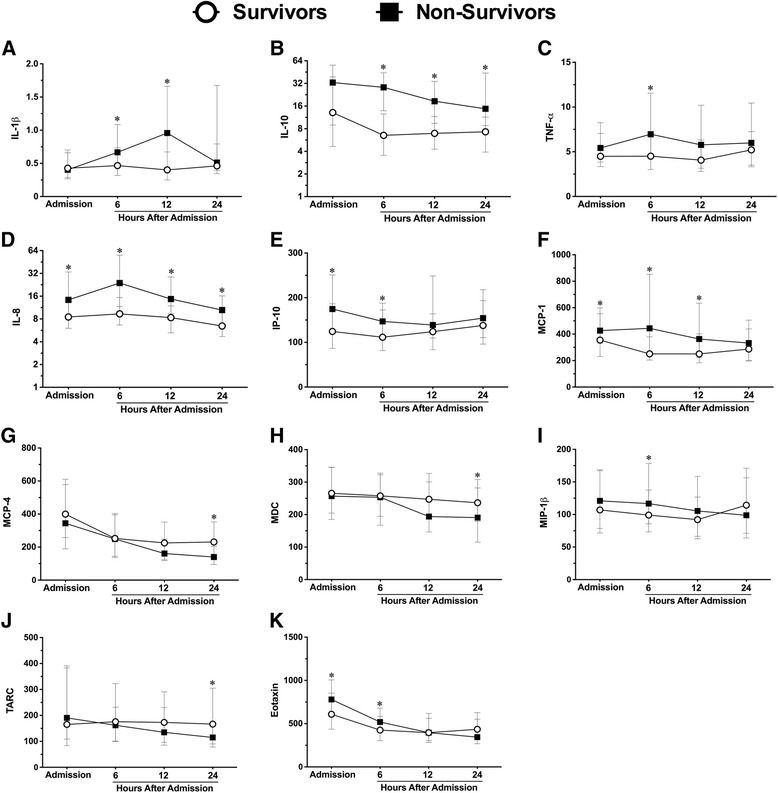
Plasma cytokine and chemokine concentrations in TBI patients stratified by mortality. Cytokines interleukin (*IL*)-1β, -10, tumor necrosis factor (*TNF*)-α (**a**–**c**). Chemokines IL-8, interferon-gamma producing protein (*IP*)-10, monocyte chemoattractant protein (*MCP*)-1, -4, macrophage-derived chemokine (*MDC*), macrophage inflammatory protein (*MIP*)-1β, thymus and activation regulated chemokine (*TARC*), and eotaxin (**d**–**k**) in TBI patients who died (*n* = 45) vs. those who lived (*n* = 119). *Lines* represent the median and interquartile range. ^*^ *p* < 0.05 vs. lived by Mann-Whitney *U* test

Alterations in inflammatory marker concentrations were observed in patients who lived vs. those who died; differences were noted in three cytokines and eight chemokines (Fig. 3a–k, respectively). IL-10 levels were elevated at 6, 12, and 24 h after admission in patients who died compared to those who lived. In non-survivors, IL-1β concentrations were 2- fold higher at 12 h (median concentrations, 0.9 vs. 0.4 pg/mL, respectively), and IL-10 concentrations were 4- fold higher at 6 h (median concentrations, 28.1 vs. 6.5 pg/mL, respectively) (Fig. 3a, b, respectively). Similar to the 6-month neurological outcome, IL-8 levels displayed the greatest elevation of all chemokines assessed in patients who died vs. those who lived (Fig. 3d). IL-8 concentrations were significantly elevated at all sampled time points, with the greatest disparity at 6 h, where patients who died had concentrations >2-fold higher than those who lived (median concentrations, 23.9 vs. 9.3 pg/mL, respectively) (Fig. 3d). Conversely, MCP-4, MDC, and TARC concentrations at 24 h after hospital admission were significantly lower in TBI patients who died compared to survivors (Fig. 3g, h, j, respectively).

### Secondary complications controlled for injury severity

#### Six-month GOSE

When controlled for admission GCS and AIS head scores, and standardized according to ranked quartiles, IL-1β (peak, 6 h; OR (odds ratio) 1.84; 95 % confidence interval (CI) 0.90–2.25), IL-10 (peak, 6 h; OR 1.76; 95 % CI 1.25–2.70), and TNF-α (peak, 6 h; OR 1.61; 95 % CI 1.13–2.29) were significant independent predictors of unfavorable 6-month outcome (GOSE 1–4) (Table 3). In addition, IL-8 (peak, admission; OR 1.66; 95 % CI 1.15–2.39) and MDC (peak, 12 h; OR 1.45; 95 % CI 1.02–2.05) were independent predictors of unfavorable outcome (Table 3).

### Mortality

Controlled for admission GCS and AIS head scores, patient death was independently associated with three cytokines and four chemokines (Table 3). IL-10 was the greatest predictor of death of all cytokines analyzed (peak, 6 h; OR 2.82; 95 % CI 1.63–4.87). However, the strongest predictor of death among chemokines as well as all evaluated inflammatory markers was IP-10 (peak, admission; OR 3.11; 95 % CI 1.83–5.27) (Table 3).

### Neurologic vs non-neurologic death

**Fig. 4:**
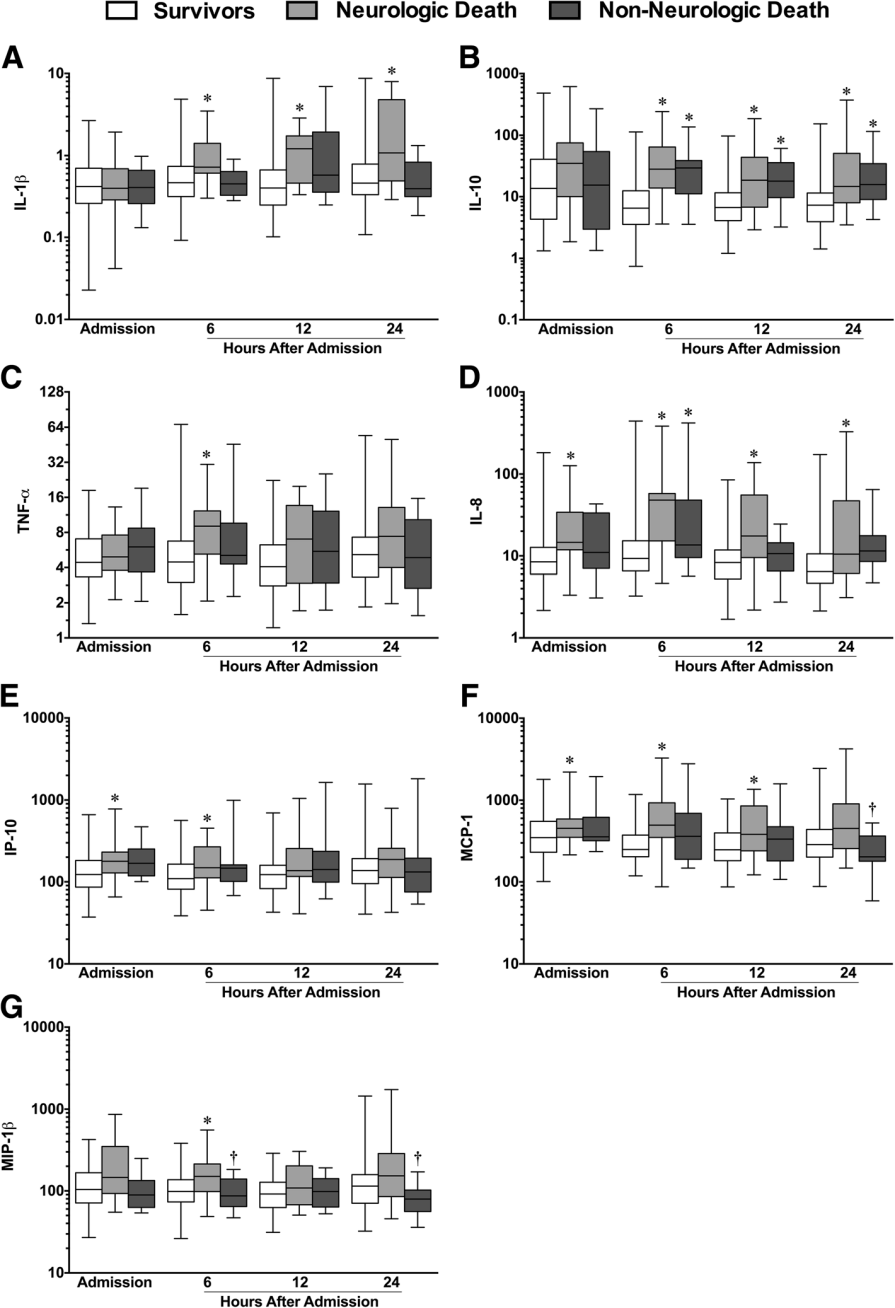
Plasma cytokine and chemokine concentrations in TBI patients according to the cause of death. I nterleukin (*IL*)-1β, -10, tumor necrosis factor (*TNF*)-α (**a**–**c**). IL-8, interferon-gamma producing protein (*IP*)-10, monocyte chemoattractant protein (*MCP*)-1, and macrophage inflammatory protein (*MIP*)-1β (**d**–**g**) in TBI patients who survived (*n* = 119*)* vs. those who died by neurologic death *(n* = 28) or by non-neurologic organ failure (*n* = 17). *Boxes* represent the median and interquartile ranges, and *whisker plot lines* represent the range. *p < 0.05 vs. patients who survived; ^†^ *p* < 0.05 vs. patients who succumbed to neurologic death, by Kruskal-Wallis

Differences in specific inflammatory biomarkers were observed between patients who lived vs. those who died by neurologic or non-neurologic organ failure. IL-1β was significantly elevated in patients who died by neurologic death vs. those who survived at 6, 12, and 24 h; no difference was found between those who died by organ failure and those who survived (Fig. 4a). IL-10 levels were elevated in patients succumbing to both neurologic and non-neurologic death compared to those who survived at all time points except admission (Fig. 4b). Similarly, IL-8 concentrations were elevated in neurologic and non-neurologic death compared to patients who died at 6 h, but were elevated only in patients succumbing to neurologic death at all other sampled time points (Fig. 4c). MCP-1 concentrations were elevated in neurologic death vs. survival at admission, 6, and 12 h, but were lower at 24 h in patients who died by non-neurologic organ failure vs. neurologic death (Fig. 4f). In addition, MIP-1β concentrations were significantly lower in patients who died by non-neurologic organ failure vs. neurologic death at 6 and 24 h (Fig. 4g).

### Combined inflammatory score and patient characteristics

A combined IS was created in a subset of 76 patients using three cytokines (IL-1β, -10, and TNF-α) and three chemokines (IL-8, MCP-1, MDC) that were associated with both unfavorable patient outcome at 6 months and mortality. Compared to patients with low a IS (<15), patients with a high IS (≥15) had significantly greater mean NE levels over 24 h from hospital admission, while Epi levels did not statistically differ (Table 4). Greater injury severity, as assessed by admission GCS, AIS head, and ISS scores, was associated with a high IS. In addition, patients with poor outcomes (unfavorable 6-month GOSE and death) had high IS scores (Table 4).

**Table 4:** Clinical characteristics according to dichotomized inflammatory scores

Characteristics	Inflammation—low (IS < 15, *n* = 33)	Inflammation—high (IS ≥ 15, *n* = 43)	*P* value
Catecholamine levels^a^ (pmol/L)			
Epi	1131.5 (656.0–1901.9)	1655.0 (733.2–4307.4)	0.110
NE	6064.3 (3081.0–18373.4)	12,110.0^*^ (7653.1–37,939.8)	0.013
Injury severity			
GCS	7.0 (4.0–9.0)	3.0^*^ (3.0–7.0)	0.002
AIS head	4.0 (3.0–5.0)	5.0^*^ (4.0–5.0)	0.006
ISS	20.5 (16.5–29.5)	29.5^*^ (20.0–35.0)	0.014
Outcome (*n*)			
Sepsis/infection	13	9	0.079
Unfavorable 6-month GOSE	14	33^*^	0.002
Overall mortality	4	16^*^	0.014
Neurologic death	2	13^*^	0.009

Values described as the median and interquartile range unless otherwise stated. IS variables consist of peak differences between unfavorable and favorable outcome over 24 h in IL-1β (6 h), IL-10 (6 h), TNF-α (6 h), IL-8 (6 h), MCP-1 (admission), and MDC (12 h)

*Abbreviations*: *IS* inflammation score, *Epi* epinephrine, *NE* norepinephrine, *GCS* Glasgow coma scale, *AIS* abbreviated injury scale, *ISS* injury severity score, *GOSE* extended Glasgow outcome scale

*p < 0.05 vs. low inflammatory score by Mann-Whitney *U* or chi-square, where appropriate

^a^Average catecholamine concentrations over 24 h

## Discussion

In this study we demonstrated that following isolated TBI, acute alterations in systemic cytokine and chemokine levels were associated with unfavorable patient outcome and death. Our sample size of 166 patients is, to our knowledge, the largest study to date to characterize the systemic inflammatory response in isolated TBI. A compelling finding of this work was the identification of a relationship between SNS activity and systemic inflammation acutely post-injury. In addition, our findings addressed the heterogeneous and complex nature of TBI which has confounded our understanding of the pathophysiology and clinical relevance of post-traumatic systemic inflammation [1]. Specifically, our study population consisted of isolated TBI patients with non-penetrating injuries, stratified according to injury severity, and sampled acutely at four time points over the first 24 h from hospital admission (Additional file 2: Table S2).

We found IL-10 was the most prominently altered of all cytokines acutely after injury, and was positively correlated with injury severity. While this is in general agreement with previous findings [21, 37, 48, 49], few studies have assessed IL-10 in isolated TBI patients [21, 49]. Furthermore, we are aware of only one previous report that evaluated IL-10 in isolated TBI patients dichotomized by injury severity, albeit in a population of 26 patients that assessed injury severity at 7 days post-injury [50]. Also, in agreement with previous research, unfavorable patient outcome and mortality were associated with elevated peripheral concentrations of IL-1β, IL-10, and TNF-α [32, 37, 46, 48]. Importantly, the relationships between cytokines and patient outcome were significant even after controlling for injury severity, suggesting a possible independent role of these mediators in secondary injury pathogenesis. Moreover, our results provide evidence that certain cytokines may be associated with specific outcomes. For example, IL-1β and TNF-α were elevated only in patients who died from neurologic death, while IL-10 was higher in patients who died as a result of both neurologic and non-neurologic organ failure. Taken together, these findings not only reinforce the globally detrimental role of IL-10 acutely after TBI, but also suggest that peripheral blood may be a viable source for biomarkers related to brain injury-specific outcomes.

Systemic concentrations of all nine chemokines analyzed were significantly different in TBI patients compared to healthy subjects, and alterations in eight of the nine chemokines were associated with poor patient outcome. In addition, we found eotaxin-3, IL-8, MCP-1, and MCP -4 were associated with injury severity. Similar to our cytokine analysis, when controlled for injury severity, seven of the nine chemokines were still related to poor patient outcome, with increases in admission IP-10 levels displaying the strongest relationship to death of all inflammatory markers analyzed. Notably, while higher IP-10 levels in patients were independently associated with mortality, on average, levels were lower in patients vs. healthy controls. The reasons for this are unclear, but it is possible that our total patient values were skewed by lower IP-10 concentrations in survivors vs. those who died. Furthermore, that a number of chemokines were lower in patients compared to controls, or lower in patients with unfavorable vs. favorable outcomes, may speak to the complex and divergent roles of chemokines in mediating secondary injury. Our findings are in general agreement with others who have identified elevations in systemic IL-8 and MCP-1 after TBI [31, 39, 44, 51, 52] and identified the relationship between this and poor patient outcome [38, 40, 45]. However, to our knowledge, no previous studies have characterized this diverse array of chemokines in the peripheral blood after human TBI. Interestingly, Helmy et al. [53] evaluated a number of cytokines and chemokines in the brain extracellular fluid in 12 TBI patients post-injury. These authors concluded that among other inflammatory mediators evaluated, IL-8, MCP-1, IP-10, and MIP-1β were elevated in the brain extracellular fluid relative to plasma, and may be centrally produced after TBI. However, this study did not evaluate plasma levels of these markers in comparison to healthy controls and subsequently, the relative systemic changes were not determined. Furthermore, Chen et al. [54] recently found serum levels of CXCL12 were significantly related to injury severity and patient death after isolated human TBI. Collectively, these recent findings and those of the current study support the deleterious involvement of chemokines beyond IL-8 and MCP-1 in secondary injury pathophysiology, and warrants further investigation.

We identified a number of specific correlations between systemic catecholamines and inflammatory mediators that are consistent with previous experimental findings. For example, Woisciechowsky et al. [24] found that SNS activation after brain trauma results in the systemic release of IL-10. The authors showed that NE and Epi signaling through peripheral blood monocyte β_2_-adrenergic receptors caused an increase in circulating IL-10 concentrations, deactivation of circulating monocytes, and subsequent immunosuppression and infection [24]. That we found a strong association between both NE, Epi, and IL-10 is consistent with the experimental findings of Woiciechowsky et al. and suggests a mechanistic role for catecholamines in mediating IL-10 production in the acute period after isolated TBI. Additionally, it has been hypothesized that IL-1β may induce SNS activation after brain trauma [55–57]. Experimental studies have shown that both CNS and peripherally injected IL-1β have the ability to stimulate the SNS [55, 58], which may then mediate leukocyte mobilization and initiate the hepatic acute phase response [15–18, 55]. This is consistent with the associations we found between IL-1β and both NE/Epi . Furthermore, we identified a positive correlation between NE, Epi, and IL-8 and MCP-1. It has been suggested that the production and release of chemokines from the liver, particularly IL-8 and MCP-1, is an important component of the systemic acute phase response after TBI [4, 16–18], particularly in mediating the mobilization and recruitment of leukocytes to the brain [17, 18, 59, 60]. In a review by Catania and colleagues [4], it was hypothesized that systemic chemokine production and release from the liver post-injury may be mediated by SNS activation and, specifically, the activation of sympathetic nerve terminals and subsequent interaction between tissue macrophages and synaptic NE [18]. In addition, NE has been found to interact with β-adrenergic receptors on cultured peripheral blood mononuclear cells to produce MCP-1 [61], and Epi has been shown in multiple studies to potentiate LPS-induced IL-8 production in monocytes [22, 62]. While the specific mechanism(s) has/have yet to be determined, the results of the current study are supportive of catecholamine- mediated chemokine production after isolated TBI.

While the pathogenesis of systemic inflammation after TBI remains uncertain, our results provide evidence consistent with a detrimental role for the innate immune system in the acute period after injury and further supports the concept that SNS hyperactivity mediates this process. Indeed, the application of an inflammation-based prognostic score showed that the net inflammatory effect seen acutely after TBI is associated with poor patient outcomes, injury severity, and significant elevations in NE. Furthermore, our findings are generally consistent with the revised interpretation of the systemic inflammatory response syndrome (SIRS) and compensatory anti-inflammatory response syndrome (CARS) noted after trauma and sepsis, which suggests that pro- and anti-inflammatory processes occur concurrently, not in a phase delayed manner as was previously hypothesized [63, 64]. We and others have consistently observed that the anti-inflammatory molecule IL-10 is among the earliest detectable mediators after trauma [48, 50, 65] and is elevated concurrently with pro-inflammatory IL-1β, IL-8, TNF-α, and numerous other chemokines in patients with poor outcomes. Notably, we did not find any association between acute inflammation and the onset of sepsis/infections. However, this study did not directly characterize cellular immune function, and previous studies have reported that immunosuppression and subsequent infection after brain trauma may be related to impaired cellular immunity, including monocyte deactivation [24, 65] and/or suppression of T cell function [66, 67].

The results of this study should be interpreted in the context of its limitations. Despite a well-controlled clinical design and moderately sized cohort of 166 TBI patients, a larger sample size would have allowed for further stratification of our patient population, particularly regarding variables such as sex, age and isolated sepsis. Also, we were only able to assess the level of inflammatory markers in patients’ blood samples for the first 24 h post-injury, and a longer sampling period may have been advantageous to assess the potential for hyperadrenergic mediated immunosuppression. In spite of these limitations, this investigation is one of the most comprehensive inflammatory studies in isolated human TBI to date. The results demonstrated a pronounced systemic cytokine/chemokine response acutely after isolated head injury and suggest that this response is associated with the degree of SNS activation.

## Conclusions

A number of peripheral cytokines and chemokines are altered in the acute period after moderate and severe isolated TBI. These alterations are associated with unfavorable patient outcomes. Moreover, the systemic inflammatory response after TBI appears to be mediated, at least in part, by a profound trauma-induced hyperadrenergic state. In the present study, we found marked increases in circulating IL-10 and alterations in all chemokines assessed within 24 h of hospital admission. Poor patient outcome was associated with alterations in IL-1β, IL-10, TNF-α, and all chemokines with the exception of eotaxin-3. Furthermore, circulating NE and Epi levels were positively correlated with IL-1β, IL-10, IL-8, eotaxin, and MCP-1. Future controlled clinical studies should continue to emphasize potential therapeutic interventions that modulate excessive SNS activation and inflammation, including treatment with selective adrenergic blocking agents.

## Additional files

Additional file 1: Table S1
**Percentage of samples analyzed within specified assay detection range**. Percentage of samples within detection limit for all circulating cytokines and chemokines analyzed. (DOCX 35 kb) Additional file 2: Table S2
**Supplement 2**. Circulating concentrations of cytokines and chemokines in all TBI patients (GCS 3–13) within 24 h of hospital admission. (DOCX 47 kb)

## References

[1] Hinson HE, Rowell S, Schreiber M (2015). Clinical evidence of inflammation driving secondary brain injury: a systematic review. J Trauma Acute Care Surg.

[2] Lu J, Goh SJ, Tng PYL, Deng YY, Ling E-A, Moochhala S (2009). Systemic inflammatory response following acute traumatic brain injury. Front Biosci.

[3] Anthony DC, Couch Y (2014). The systemic response to CNS injury. Exp Neurol.

[4] Catania A, Lonati C, Sordi A, Gatti S (2009). Detrimental consequences of brain injury on peripheral cells. Brain Behav Immun.

[5] Morganti-Kossmann MC, Rancan M, Stahel PF, Kossmann T (2002). Inflammatory response in acute traumatic brain injury: a double-edged sword. Curr Opin Crit Care.

[6] Bergold PJ (2016). Treatment of traumatic brain injury with anti-inflammatory drugs. Exp Neurol.

[7] Desborough JP (2000). The stress response to trauma and surgery. Br J Anaesth.

[8] Hörtnagl DDH, Hammerle AF, Hackl JM, Brücke T, Rumpl E, Hörtnagl H (1980). The activity of the sympathetic nervous system following severe head injury. Intensive Care Med.

[9] Clifton GL, Robertson CS, Kyper K, Taylor AA, Dhekne RD, Grossman RG (1983). Cardiovascular response to severe head injury. J Neurosurg.

[10] Clifton GL, Ziegler MG, Grossman RG (1981). Circulating catecholamines and sympathetic activity after head injury. Neurosurgery.

[11] Hamill RW, Woolf PD, McDonald JV, Lee LA, Kelly M (1987). Catecholamines predict outcome in traumatic brain injury. Ann Neurol.

[12] Woolf PDP, Hamill RWR, Lee LAL, McDonald JVJ (1988). Free and total catecholamines in critical illness. Am J Physiol.

[13] Woolf PD, Hamill RW, Lee LA, Cox C, McDonald JV (1987). The predictive value of catecholamines in assessing outcome in traumatic brain injury. J Neurosurg.

[14] Da Luz LT, Capone Neto A, DaCosta LD, Inaba K, Rhind SG, Nascimento B (2015). Catecholamines as outcome markers in traumatic brain injury. Can J Surg.

[15] Wilcockson DC, Campbell SJ, Anthony DC, Perry VH (2002). The systemic and local acute phase response following acute brain injury. J Cereb Blood Flow Metab.

[16] Campbell SJ, Perry VH, Pitossi FJ, Butchart AG, Chertoff M, Waters S (2005). Central nervous system injury triggers hepatic CC and CXC chemokine expression that is associated with leukocyte mobilization and recruitment to both the central nervous system and the liver. Am J Pathol.

[17] Campbell SJ, Wilcockson DC, Butchart AG, Perry VH, Anthony DC (2002). Altered chemokine expression in the spinal cord and brain contributes to differential interleukin-1beta-induced neutrophil recruitment. J Neurochem.

[18] Campbell SJ, Hughes PM, Iredale JP, Wilcockson DC, Waters S, Docagne F (2003). CINC-1 is an acute-phase protein induced by focal brain injury causing leukocyte mobilization and liver injury. FASEB J.

[19] Li M, Li F, Luo C, Shan Y, Zhang L, Qian Z (2011). Immediate splenectomy decreases mortality and improves cognitive function of rats after severe traumatic brain injury. J Trauma.

[20] Chu W, Li M, Li F, Hu R, Chen Z, Lin J (2013). Immediate splenectomy down-regulates the MAPK-NF-κB signaling pathway in rat brain after severe traumatic brain injury. J Trauma Acute Care Surg.

[21] Shimonkevitz R, Bar-Or D, Harris L, Dole K, McLaughlin L, Yukl R (1999). Transient monocyte release of interleukin-10 in response to traumatic brain injury. Shock.

[22] van der Poll T, Lowry SF (1997). Lipopolysaccharide-induced interleukin 8 production by human whole blood is enhanced by epinephrine and inhibited by hydrocortisone. Infect Immun.

[23] van der Poll T, Coyle SM, Barbosa K, Braxton CC, Lowry SF (1996). Epinephrine inhibits tumor necrosis factor-alpha and potentiates interleukin 10 production during human endotoxemia. J Clin Invest.

[24] Woiciechowsky C, Asadullah K, Nestler D, Eberhardt B, Platzer C, Schöning B (1998). Sympathetic activation triggers systemic interleukin-10 release in immunodepression induced by brain injury. Nat Med.

[25] Bierhaus A, Wolf J, Andrassy M, Rohleder N, Humpert PM, Petrov D (2003). A mechanism converting psychosocial stress into mononuclear cell activation. Proc Natl Acad Sci U S A.

[26] Schroeppel TJ, Fischer PE, Zarzaur BL, Magnotti LJ, Clement LP, Fabian TC (2010). Beta-adrenergic blockade and traumatic brain injury: protective?. J Trauma Acute Care Surg.

[27] Schroeppel TJ, Sharpe JP, Magnotti LJ, Weinberg JA, Clement LP, Croce MA (2014). Traumatic brain injury and β-blockers: not all drugs are created equal. J Trauma Acute Care Surg.

[28] Xu L, Yu W-K, Lin Z-L, Tan S-J, Bai X-W, Ding K (2015). Impact of β-adrenoceptor blockade on systemic inflammation and coagulation disturbances in rats with acute traumatic coagulopathy. Med Sci Monit.

[29] Rough J, Engdahl R, Opperman K, Yerrum S, Monroy MA, Daly JM (2009). β2 adrenoreceptor blockade attenuates the hyperinflammatory response induced by traumatic injury. Surgery.

[30] Ley EJ, Clond MA, Bukur M, Park R, Chervonski M, Dagliyan G (2012). β-Adrenergic receptor inhibition affects cerebral glucose metabolism, motor performance, and inflammatory response after traumatic brain injury. J Trauma Acute Care Surg.

[31] Maier B, Schwerdtfeger K, Mautes A, Holanda M, Müller M, Steudel WI (2001). Differential release of interleukines 6, 8, and 10 in cerebrospinal fluid and plasma after traumatic brain injury. Shock.

[32] Taşçi A, Okay O, Gezici AR, Ergün R, Ergüngör F (2003). Prognostic value of interleukin-1 beta levels after acute brain injury. Neurol Res.

[33] Chiaretti A, Genovese O, Aloe L, Antonelli A, Piastra M, Polidori G (2005). Interleukin 1β and interleukin 6 relationship with paediatric head trauma severity and outcome. Childs Nerv Syst.

[34] Venetsanou K, Vlachos K, Moles A, Fragakis G, Fildissis G, Baltopoulos G (2007). Hypolipoproteinemia and hyperinflammatory cytokines in serum of severe and moderate traumatic brain injury (TBI) patients. Eur Cytokine Netw.

[35] Goodman JC, Robertson CS, Grossman RG, Narayan RK (1990). Elevation of tumor necrosis factor in head injury. J Neuroimmunol.

[36] Santarsieri M, Kumar RG, Kochanek PM, Berga S, Wagner AK (2015). Variable neuroendocrine-immune dysfunction in individuals with unfavorable outcome after severe traumatic brain injury. Brain Behav Immun.

[37] Schneider Soares FM, Menezes de Souza N, Libório Schwarzbold M, Paim Diaz A, Costa Nunes J, Hohl A (2012). Interleukin-10 is an independent biomarker of severe traumatic brain injury prognosis. Neuroimmunomodulation.

[38] Gopcevic A, Mazul-Sunko B, Marout J, Sekulic A, Antoljak N, Siranovic M (2007). Plasma interleukin-8 as a potential predictor of mortality in adult patients with severe traumatic brain injury. Tohoku J Exp Med.

[39] Rhodes J, Sharkey J, Andrews P (2009). Serum IL-8 and MCP-1 concentration do not identify patients with enlarging contusions after traumatic brain injury. J Trauma.

[40] Kushi H, Saito T, Makino K, Hayashi N (2003). IL-8 is a key mediator of neuroinflammation in severe traumatic brain injuries. Acta Neurochir Suppl.

[41] Stein DM, Lindell A, Murdock KR, Kufera JA, Menaker J, Keledjian K (2011). Relationship of serum and cerebrospinal fluid biomarkers with intracranial hypertension and cerebral hypoperfusion after severe traumatic brain injury. J Trauma.

[42] Dziurdzik P, Krawczyk L, Jalowiecki P, Kondera-Anasz Z, Menon L (2004). Serum interleukin-10 in ICU patients with severe acute central nervous system injuries. Inflamm Res.

[43] Jaerve A, Müller HW (2012). Chemokines in CNS injury and repair. Cell Tissue Res.

[44] Buonora JE, Yarnell AM, Lazarus RC, Mousseau M, Latour LL, Rizoli SB (2015). Multivariate analysis of traumatic brain injury: development of an assessment score. Front Neurol.

[45] Di Battista AP, Buonora JE, Rhind SG, Hutchison MG, Baker AJ, Rizoli SB (2015). Blood biomarkers in moderate-to-severe traumatic brain injury: potential utility of a multi-marker approach in characterizing outcome. Front Neurol.

[46] Kumar RG, Boles JA, Wagner AK (2015). Chronic inflammation after severe traumatic brain injury: characterization and associations with outcome at 6 and 12 months postinjury. J Head Trauma Rehabil.

[47] Grund B, Sabin C (2010). Analysis of biomarker data: logs, odds ratios, and receiver operating characteristic curves. Curr Opin HIV AIDS.

[48] Ferreira LCB, Regner A, Miotto KDL, de Moura S, Ikuta N, Vargas AE (2014). Increased levels of interleukin-6, -8 and -10 are associated with fatal outcome following severe traumatic brain injury. Brain Inj.

[49] Hensler T, Sauerland S, Riess P, Hess S, Helling HJ, Andermahr J (2000). The effect of additional brain injury on systemic interleukin (IL)-10 and IL-13 levels in trauma patients. Inflamm Res.

[50] Woiciechowsky C, Schöning B, Cobanov J, Lanksch WR, Volk H-D, Döcke W-D (2002). Early IL-6 plasma concentrations correlate with severity of brain injury and pneumonia in brain-injured patients. J Trauma.

[51] Mussack T, Biberthaler P, Kanz K-G, Wiedemann E, Gippner-Steppert C, Mutschler W (2002). Serum S-100B and interleukin-8 as predictive markers for comparative neurologic outcome analysis of patients after cardiac arrest and severe traumatic brain injury. Crit Care Med.

[52] Sohrevardi SM, Ahmadinejad M, Said K, Sarafzadeh F, Zadeh SS, Yousefi M (2013). Evaluation of TGF β1, IL-8 and nitric oxide in the serum of diffuse axonal injury patients and its association with clinical status and outcome. Turk Neurosurg.

[53] Helmy A, De Simoni M-G, Guilfoyle MR, Carpenter KLH, Hutchinson PJ (2011). Cytokines and innate inflammation in the pathogenesis of human traumatic brain injury. Prog Neurobiol.

[54] Chen T-J, Wu W-Q, Ying G-R, Fu Q-Y, Xiong K (2015). Serum CXCL12 concentration in patients with severe traumatic brain injury are associated with mortality. Clin Chim Acta.

[55] Kenney MJ, Blecha F, Wang Y, McMurphy R, Fels RJ (2002). Sympathoexcitation to intravenous interleukin-1β is dependent on forebrain neural circuits. Am J Physiol Heart Circ Physiol.

[56] Saindon CS, Blecha F, Musch TI, Morgan DA, Fels RJ, Kenney MJ (2001). Effect of cervical vagotomy on sympathetic nerve responses to peripheral interleukin-1beta. Auton Neurosci.

[57] Woiciechowsky C, Volk HD (2005). Increased intracranial pressure induces a rapid systemic interleukin-10 release through activation of the sympathetic nervous system. Acta Neurochir Suppl.

[58] Woiciechowsky C, Schöning B, Daberkow N, Asche K, Lanksch WR, Döcke WD (1999). Brain IL-1beta increases neutrophil and decreases lymphocyte counts through stimulation of neuroimmune pathways. Neurobiol Dis.

[59] Campbell SJ, Jiang Y, Davis AEM, Farrands R, Holbrook J, Leppert D (2007). Immunomodulatory effects of etanercept in a model of brain injury act through attenuation of the acute-phase response. J Neurochem.

[60] Giles JA, Greenhalgh AD, Davies CL, Denes A, Shaw T, Coutts G (2015). Requirement for interleukin-1 to drive brain inflammation reveals tissue-specific mechanisms of innate immunity. Eur J Immunol.

[61] Takahashi H, Tsuda Y, Kobayashi M, Herndon DN, Suzuki F (2004). Increased norepinephrine production associated with burn injuries results in CCL2 production and type 2 T cell generation. Burns.

[62] Kavelaars A, van de Pol M, Zijlstra J, Heijnen CJ (1997). Beta 2-adrenergic activation enhances interleukin-8 production by human monocytes. J Neuroimmunol.

[63] Gentile LF, Cuenca AG, Efron PA, Ang D, Bihorac A, McKinley BA (2012). Persistent inflammation and immunosuppression: a common syndrome and new horizon for surgical intensive care. J Trauma Acute Care Surg.

[64] Osuchowski MF, Welch K, Siddiqui J, Remick DG (2006). Circulating cytokine/inhibitor profiles reshape the understanding of the SIRS/CARS continuum in sepsis and predict mortality. J Immunol.

[65] Rizoli SB, Rhind SG, Shek PN, Inaba K, Filips D, Tien H (2006). The immunomodulatory effects of hypertonic saline resuscitation in patients sustaining traumatic hemorrhagic shock. Ann Surg.

[66] Quattrocchi KB, Frank EH, Miller CH, MacDermott JP, Hein L, Frey L (1990). Suppression of cellular immune activity following severe head injury. J Neurotrauma.

[67] Wolach B, Sazbon L, Gavrieli R, Broda A, Schlesinger M (2001). Early immunological defects in comatose patients after acute brain injury. J Neurosurg.

