# Supplementary material for: Inflammatory cytokine and chemokine profiles are associated with patient outcome and the hyperadrenergic state following acute brain injury
Source: J Neuroinflammation. 2016 Feb 16;13:40. doi: 10.1186/s12974-016-0500-3 (PMC4754875; doi:10.1186/s12974-016-0500-3)
Supplement: Additional file 1: Table S1 — Percentage of samples analyzed within specified assay detection range. Percentage of samples within detection limit for all circulating cytokines and chemokines analyzed. (DOCX 35 kb) [file 12974_2016_500_MOESM1_ESM.docx]

### Additional file 1 – Percentage of samples analyzed within specified assay detection range.

**Table S1.** Percentage of samples within detection limit for all circulating cytokines and chemokines analyzed.

|  |  |  | **Hours After Admission** | | |
| --- | --- | --- | --- | --- | --- |
| **Markers** | **Healthy (n = 21)** | **Admission (n = 157)** | **6 (n = 148)** | **12 (n = 143)** | **24 (n = 138)** |
| *Cytokines (pg/mL)* | | | | | |
| IFN-γ | 9 (42.9) | 54 (34.4) | 48 (32.4) | 44 (30.8) | 46 (33.3) |
| IL-1β | 7 (33.3) | 86 (54.8) | 87 (58.8) | 95 (66.4) | 87 (63.0) |
| IL-2 | 9 (42.9) | 57 (36.3) | 55 (37.2) | 68 (47.5) | 60 (43.5) |
| IL-4 | 0 (0.0) | 20 (12.7) | 18 (12.2) | 21 (14.7) | 18 (13.0) |
| IL-5 | 17 (80.9) | 101 (64.3) | 106 (71.6) | 93 (65.0) | 87 (63.0) |
| IL-10 | 19 (90.5) | 156 (99.4) | 147 (99.3) | 141 (98.6) | 134 (97.1) |
| IL-12p70 | 15 (71.4) | 50 (31.8) | 38 (25.7) | 40 (28.0) | 42 (30.4) |
| IL13 | 9 (42.9) | 36 (22.9) | 35 (23.6) | 23 (16.1) | 23 (16.7) |
| TNF-α | 20 (95.2) | 154 (98.1) | 147 (99.3) | 143 (100) | 137 (99.3) |
| *Chemokines (pg/mL)* | | | | | |
| Eotaxin | 21 (100) | 133 (84.7) | 130 (87.8) | 129 (90.2) | 125 (90.6) |
| Eotaxin-3 | 11 (52.4) | 111 (70.7) | 95 (64.2) | 94 (65.7) | 80 (58.0) |
| IL-8 | 20 (95.2) | 148 (94.3) | 147 (99.3) | 141 (98.6) | 138 (100) |
| IP-10 | 21 (100) | 148 (94.3) | 148 (100) | 142 (99.3) | 138 (100) |
| MCP-1 | 21 (100) | 149 (94.9) | 148 (100) | 141 (98.6) | 138 (100) |
| MCP-4 | 21 (100) | 149 (94.9) | 147 (99.3) | 142 (99.3) | 137 (99.3) |
| MDC | 20 (95.2) | 146 (93.0) | 147 (99.3) | 141 (98.6) | 138 (100) |
| MIP-1β | 21 (100) | 149 (94.9) | 148 (100) | 142 (99.3) | 138 (100) |
| TARC | 21 (100) | 149 (94.9) | 146 (98.6) | 142 (99.3) | 136 (98.5) |

Abbreviations: TBI, traumatic brain injury; GCS, Glasgow coma scale; IFN-γ, interferon gamma; IL, interleukin; TNF-α, tumor necrosis factor - alpha; IP-10, interferon-gamma induced protein - 10; MCP, monocyte chemoattractant protein; MDC, macrophage-derived chemokine; MIP-1β, macrophage inflammatory protein – 1 beta; TARC, thymus and activation regulated chemokine.

Data are presented as the number and percent, n (%), of total available blood samples at each time point within the detection range for the assay, and with a coefficient of variance (CV) less than 25%.
