# Supplementary material for: Inflammatory cytokine and chemokine profiles are associated with patient outcome and the hyperadrenergic state following acute brain injury
Source: J Neuroinflammation. 2016 Feb 16;13:40. doi: 10.1186/s12974-016-0500-3 (PMC4754875; doi:10.1186/s12974-016-0500-3)
Supplement: Additional file 2: Table S2 — Supplement 2. Circulating concentrations of cytokines and chemokines in all TBI patients (GCS 3–13) within 24 h of hospital admission. (DOCX 47 kb) [file 12974_2016_500_MOESM2_ESM.docx]

**Table S2.** Circulating concentrations of cytokines and chemokines in all TBI patients (GCS 3-13) within 24 hours of hospital admission.

|  |  |  | **Hours After Admission** | | |
| --- | --- | --- | --- | --- | --- |
| **Markers** | **Healthy** | **Admission** | **6** | **12** | **24** |
| *Cytokines (pg/mL)* | | | | | |
| IFN-γ | ND | ND | ND | ND | ND |
| IL-1β | ND | 0.4 (0.0 – 2.7) | 0.5 (0.1 – 4.9) | 0.4 (0.1 – 3.9) | 0.5 (0.1 – 8.7) |
| IL-2 | ND | ND | ND | ND | ND |
| IL-4 | ND | ND | ND | ND | ND |
| IL-5 | 0.4 (0.1 – 2.2) | 0.5 (0.1 – 6.7) | 0.6 (0.1 – 6.5) | 0.6 (0.0 – 4.9) | 0.6 (0.0 – 3.9) |
| IL-10 | 1.7 (1.4 – 3.9) | 14.4^*^ (0.4 – 617.6) | 8.2^*^ (0.7 – 136.9) | 8.1^*^ (1.2 – 120.7) | 7.9^*^ (1.4 – 153.2) |
| IL-12p70 | 0.9 (0.1 – 4.3) | ND | ND | ND | ND |
| IL13 | ND | ND | ND | ND | ND |
| TNF-α | 3.9 (2.8 – 5.6) | 4.9 (1.3 – 19.2) | 5.0 (1.6 – 67.8) | 4.3 (1.2 – 25.4) | 5.2 (1.5 – 54.3) |
| *Chemokines (pg/mL)* | | | | | |
| Eotaxin | 590.2 (335.6 – 949.4) | 628.7 (234.2 – 2273.0) | 446.2 (151.8 – 1448.0) | 388.2^*^ (78.4 – 1243.0) | 405.9^*^ (149.2 – 1785.0) |
| Eotaxin-3 | 5.1 (3.6 – 9.4) | 9.5 ^*^ (2.5 – 28.0) | 8.7^*^ (2.6 – 34.0) | 8.3^*^ (2.2 – 49.6) | 8.8^*^ (3.0 – 32.2) |
| IL-8 | 3.5 (1.6 – 12.4) | 10.0^*^ (2.2 – 182.9) | 10.7^*^ (3.2 – 168.3) | 9.3^*^ (1.7 – 137.9) | 6.9^*^ (2.1 – 173.5) |
| IP-10 | 175.6 (102.1 – 283.9) | 137.6 (37.4 – 779.3) | 119.5^*^ (38.7 – 994.3) | 129.5^*^ (41.0 – 1641.0) | 138.3 (40.6 – 1821.0) |
| MCP-1 | 235.4 (143.5 – 335.4) | 378.7^*^ (101.1 – 2215.0) | 283.5 (87.3 – 3838.0) | 267.8 (86.9 – 1588.0) | 290.5 (58.7 – 3077.0) |
| MCP-4 | 295.6 (107.3 – 587.8) | 386.7 (71.0 – 1727.0) | 252.5 (53.3 – 1423.0) | 200.3 (24.3 – 1345.0) | 203.9 (27.5 – 1148.0) |
| MDC | 321.9 (201.8 – 593.4) | 259.9 ^*^ (41.2 – 791.8) | 253.5^*^ (39.3 – 547.3) | 237.4^*^ (67.3 – 664.8) | 228.6^*^ (57.3 – 787.4) |
| MIP-1β | 62.0 (37.0 – 97.9) | 110.8^*^ (27.1 – 519.6) | 102.7^*^ (26.4 – 555.7) | 96.5^*^ (31.3 – 304.7) | 111.7^*^ (32.4 – 943.9) |
| TARC | 100.7 (34.8 – 314.0) | 171.8^*^ (36.3 – 1584.0) | 172.8^*^ (32.6 – 1295.0) | 167.0^*^ (42.0 – 978.7) | 140.1^*^ (30.0 – 959.7) |

Abbreviations: TBI, traumatic brain injury; GCS, Glasgow coma scale; IFN-γ, interferon gamma; IL, interleukin; TNF-α, tumor necrosis factor - alpha; IP-10, interferon-gamma induced protein - 10; MCP, monocyte chemoattractant protein; MDC, macrophage-derived chemokine; MIP-1β, macrophage inflammatory protein – 1 beta; TARC, thymus and activation regulated chemokine.

Data are presented as median (range).

* = adjusted *P* < 0.05 vs. healthy control subjects.

ND = >50% of the samples below the assay detection level.
